# Lack of correlation of *Brucella* antibody titers with clinical outcomes and culture positivity of brucellosis

**DOI:** 10.1186/s40794-021-00130-w

**Published:** 2021-02-02

**Authors:** Shahad A. Alsubaie, Shouq A. Turkistani, Alanoud A. Zeaiter, Abrar K. Thabit

**Affiliations:** Pharmacy Practice Department, Faculty of Pharmacy, King Abdulaziz University, 7027 Abdullah Al-Sulaiman Rd, Jeddah, 22254-2265 Saudi Arabia

**Keywords:** Antibody, *Brucella*, Brucellosis, Culture, Serology, Titer

## Abstract

**Background:**

Brucellosis is a zoonotic disease caused by *Brucella* spp., namely *B. melitensis* and *B. abortus* in humans. Culturing is the gold standard method for diagnosis; however, because *Brucella* is a slow-growing bacterium, which may delay diagnosis, other faster methods, such as serology, are used. Studies on the correlation between *Brucella* antibody titers and clinical outcomes are limited. Therefore, this study assessed such correlation and evaluated the correlation between baseline serological results with culture positivity and clinical picture.

**Methods:**

Patients tested positive for *Brucella* antibodies at baseline and diagnosed with brucellosis between January 2008 and December 2018 were included. Collected data included clinical outcomes, baseline culture positivity (growth in culture), arthralgia, baseline and end of therapy (EOT) temperature, white blood cell count, C-reactive protein level, and erythrocyte sedimentation rate.

**Results:**

Of 695 patients tested for *Brucella* antibodies, only 94 had positive baseline serology and diagnosed with acute brucellosis, among whom 63 had EOT serology. No significant correlations were found between EOT antibody titers of both *Brucella* spp. and clinical cure, mortality, length of stay, and duration of therapy. Additionally, no correlations were found between baseline serology and culture positivity, arthralgia, temperature, and other lab values.

**Conclusion:**

*Brucella* serology does not correlate with clinical outcomes at EOT nor with culture positivity at baseline. Therefore, healthcare providers are advised to consider the whole clinical picture of a brucellosis patient without relying solely on serological results during follow up and not replace culturing with serology testing alone at the time of diagnosis.

## Introduction

*Brucella* spp. is a Gram-negative coccobacillus that causes brucellosis (also known as “Malta fever”), a contagious zoonotic infectious disease [1]. The genus *Brucella* consists of several species, four of which causes a disease in humans. The two most common species include *B*. *melitensis which is transmitted from goats and sheep followed by B*. *abortus that comes from cows* [2, 3]*.* The infection usually starts with nonspecific symptoms, such as fatigue, headache, dry cough, and night sweats [2]. However, in severe cases it can be associated with variable complications, such as cardiac, neurologic, bone and joint, ophthalmic, or testicular involvement [2]. Brucellosis is an endemic disease in several countries, including Saudi Arabia, Iraq, Jordan, and other Middle East countries, as well as Turkey, central Asia, Mexico, South America, and Asia-Pacific region [4, 5].

To aid the clinical diagnosis of brucellosis, direct and indirect laboratory methods are used. Direct methods include microbiological culturing and the detection of particular *Brucella* genes using polymerase chain reaction (PCR) test [1]. Brucella culture is considered the gold standard and provides the definitive diagnosis of the disease [6]. However, as *Brucella* is a slow-growing bacterium, it may take the culture a week or longer to show growth [7]. Additionally, patients with a long-standing disease may have negative culture results due to bacterial eradication without complete clinical recovery. Therefore, indirect serological assays are used alternatively and provide faster results. The Rose Bengal testing, tube agglutination, and enzyme-linked immunosorbent assay (ELISA) are the most recommended methods of *Brucella* serology [1]. Moreover, some nonspecific laboratory tests, such as white blood cell (WBC) count, C-reactive protein (CRP), erythrocyte sedimentation rate (ESR), serum lactate dehydrogenase, and alkaline phosphatase maybe elevated [7].

In a response to an infection with *Brucella* spp., the immune system naturally produces antibodies. IgM isotype is first produced followed by the IgG and IgA isotypes. With appropriate treatment, the titer of these antibodies should gradually fall off. The remaining high titer of IgG and IgA indicate a possibility of relapse or progression to a chronic focal disease [2].

A retrospective study of acute brucellosis patients showed that *Brucella* antibodies remained persistently positive even after the patients demonstrated full clinical recovery [8]. As serological cure of brucellosis is defined when antibody titers reach less than 1:320, the rate of serological cure in this study increased from 8.3% in the first 3 months to 71.4% after two or more years. Besides this study, no other studies addressing the relationship between *Brucella* antibody titers and clinical outcomes were found in the literature. As such, this study aims to evaluate such correlation, as well as to assess the correlation of baseline serology with baseline culture positivity and clinical picture in an area of disease endemicity. It is hypothesized that follow up serology does not correlate with clinical outcomes, whereas culture positivity correlates with high antibody titers.

## Materials and methods

### Study design and patients

This was a retrospective, cross-sectional study of acute brucellosis patients admitted to King Abdulaziz University Hospital in Jeddah, Saudi Arabia during the period from January 2008 to December 2018. Ethical approval was obtained from the Biomedical Research Ethics Unit, Faculty of Medicine, King Abdulaziz University. Patients aged 18 years or older who had positive *Brucella* serology (regardless of the titer), diagnosed with acute brucellosis based on serology (defined as antibody titer of ≥1:640), positive *Brucella* culture, or clinical picture, and received antibiotic treatment were included in the study. Patients who did not receive treatment for brucellosis despite positive serology (of < 1:640) were excluded as such patients did not exhibit clinical signs and symptoms of brucellosis to be diagnosed with the disease at baseline. Hence, no antibiotic therapy was warranted.

### Laboratory tests

The serological test used in our hospital is the standard tube agglutination test (SAT) which measures total antibodies (IgG and IgM) of *B. melitensis* and *B. abortus* and provides semi-quantitative results (titers) [9]. Two types of antigens are used in the test, one for *B. melitensis* and the other for *B. abortus*. The tubes are incubated at 37 °C for up to 48 h after the suspension is mixed with serum. Positive and negative controls provided with the test kit were used to validate the test. The reported sensitivity and specificity of SAT are 95.6–100% and 96–100%, respectively [10, 11]. Due to the endemic nature of brucellosis in our country, an antibody titer of at least 1:640 of either *Brucella* spp. is needed to confirm the serological diagnosis, especially in the absence of positive *Brucella* culture or classic clinical symptoms suggestive of acute brucellosis.

Microbiological diagnosis for brucellosis is established after blood samples labelled with “*Brucella*” are collected in BD BACTEC™ blood culture media bottles (Beckton, Dickinson and Company, NJ, USA) and incubated in BD BACTEC™ FX system at 37 °C for up to 14 days. When the system generates an alarm, the positive sample is subcultured on blood and chocolate agar plates, which are incubated in 5–10% CO_2_ for 24–48 h. Once growth is observed on the plate, the bacteria are identified via the morphology of the colonies, Gram staining, and biochemical testing using urease and oxidase (*Brucella* produce both). This process provides identification to the genus level. According to the microbiology lab protocol, species identification is no longer carried out using Matrix-Assisted Laser Desorption/Ionization-Time Of Flight (MALDI-TOF) mass spectrometry due to biohazard risk. Alternatively, species are identified using serology.

### Correlation parameters

The correlation of *Brucella* antibody titers at end of therapy (EOT) was assessed with clinical cure (defined as resolution of clinical signs and symptoms including resolution of fever and normalization of WBC count), mortality, length of stay, duration of therapy, and EOT temperature, WBC count, CRP level, and ESR. Moreover, correlation of baseline *Brucella* serological results was assessed with *Brucella* culture positivity (growth in culture), baseline temperature, WBC count, CRP level, and ESR. A serological cure cutoff value was set at < 1:320 based on previous data [8].

### Statistical analysis

Descriptive statistics using percentages, mean ± standard deviation for normally distributed data, and median (interquartile range, IQR) for non-normally distributed data were used. Normal distribution was determined using Shapiro-Wilk test for normality. Pearson’s correlation was used to assess the correlation between *Brucella* antibody titers with different outcomes at EOT and baseline. Two-tailed statistical significance was indicated by a *P* value of < 0.05. SPSS version 24.0 (SPSS, Inc., Chicago, Illinois, USA) was used for statistical analysis.

## Results

### Patients

Out of 695 patients tested for *Brucella* via serology, 94 had positive serology and were diagnosed with acute brucellosis. Table 1 lists the characteristics of patients included in the study. More than half of the patients were males with an average age of 48 ± 19 years. The majority were whites of Arab descent. Overall, patients did not have fever or leukocytosis at baseline; though, CRP levels and ESR were mostly elevated. In addition, less than one-third of the patients had complicated brucellosis or arthralgia.

**Table 1:** Baseline characteristics

Characteristic	All Patients (***n*** = 94)
Age, years	48 ± 19
Sex, male, n (%)	58 (61.7)
Race, n (%)	
• White	83 (88.3)
• Black	3 (3.2)
• Asian	8 (8.5)
Location, n (%)	
• Outpatient	43 (45.7)
• Inpatient medical ward	49 (52.1)
• ICU	2 (2.1)
Charlson co-morbidity index	1.9 ± 2.1
Baseline *B. melitensis* antibody titer^a^	> 1:1280 [1:1280- > 1:1280]
Baseline *B. melitensis* antibody titer ≥1:640, n (%)	78 (83)
Baseline *B. abortus* antibody titer^a^	> 1:1280 [1:1280- > 1:1280]
Baseline *B. abortus* antibody titer ≥1:640, n (%)	73 (77.7)
Baseline temperature, °C	36.8 [36–37.3]
Baseline white blood cells count, cells/mm^3^	6.6 ± 4.9
Baseline C-reactive protein, mg/L	34.5 ± 39.9
Baseline erythrocyte sedimentation rate, mm/hr	29 ± 23.5
Duration of therapy^a^	45 [45–135]
Risk factors for infection, n (%)	
• Unpasteurized dairy product	42 (44.7)
• Direct contact with animals	4 (4.3)
• Both	8 (8.5)
• Other	1 (1)
• Unknown	39 (41.5)
Diagnostic test positivity, n (%)	
• *Brucella* serology alone	42 (44.7)
• Both *Brucella* serology and culture	52 (55.3)
*Brucella* spp., n (%)	
• *B. melitensis*	4 (4.3)
• *B. abortus*	2 (2.1)
• Both	88 (93.6)
Complicated brucellosis, n (%)	29 (30.9)
• Spondylodiscitis	17 (59)
• Neurobrucellosis	5 (17)
• Orchitis	3 (10.3)
• Other	3 (10.3)
• > 1 organ	1 (3.4)
Arthralgia, n (%)	30 (31.9)
Regimen used, n (%)	
• Aminoglycoside + doxycycline + rifampin	44 (46.8)
• Doxycycline + rifampin	34 (36.2)
• Other	16 (17)

Data are presented as mean ± SD unless specified otherwise

^a^ Median [IQR]

EOT outcomes are presented in Table 2. Sixty three of the 94 patients included in the study had EOT serological results available. Median antibody titers at EOT were lower than their baseline counterparts. Similarly, median CRP level and ESR showed a decrease from baseline. Most patients experienced clinical cure and only 5 died (one patient died due to septic shock and pulmonary embolism and another due to an underlying liver disease; both unrelated to brucellosis. The remaining three patients died due to unknown causes).

**Table 2:** End of therapy outcomes

Outcome	All Patients (***n*** = 94)
Length of stay, days^a, b^	9 [5–17]
Clinical cure, n (%)	80 (85.1)
Mortality, n (%)	5 (5.3)
*B. melitensis* antibody titer^a, c^	1:320 1:320–1:640
*B. melitensis* antibody titer < 1:320, n (%)^c^	28 (29.8)
*B. abortus* antibody titer^a, c^	1:320 1:160–1:640
*B. abortus* antibody titer < 1:320, n (%)^c^	33 (35.1)
Temperature, °C^a^	36.7 [36.5–36.9]
white blood cells count, cells/mm^3^	6.2 ± 3.4
C-reactive protein, mg/L^a^	3.2 [3.1–3.3]
erythrocyte sedimentation rate, mm/hr^a^	8 [5–22]

Data are presented as mean ± SD unless specified otherwise

^a^ Median [IQR]

^b^ Data for hospitalized patients only

^c^ Only 63 of patients had end of therapy serology and hence the percentage reflects this population

### Correlation with EOT parameters

**Fig. 1:**
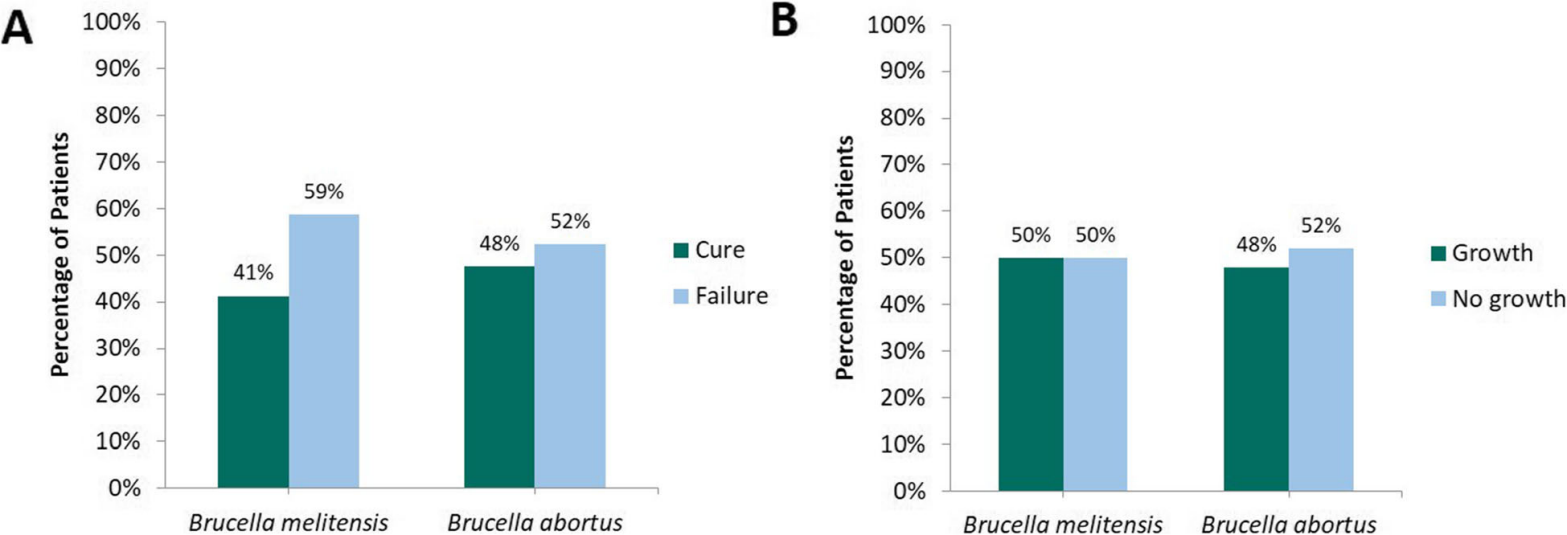
**a** Distribution of clinical outcomes in patients with end of therapy antibody titers of < 1:320 and **b** distribution of culture results in patients with baseline antibody titers of ≥1:640

The correlations between EOT *Brucella* antibody titers with different outcome variables are shown in Table 3. A significant, though weak, correlation between clinical cure and low EOT antibody titers of both *Brucella* spp. was found (R = 0.29 and 0.28; *P* = 0.03) indicating a rejection of the null hypothesis. Nevertheless, this correlation was lost when antibody titers were stratified based on the serological cure cutoff value of < 1:320 (*P* ≥ 0.05). This is demonstrated in Fig. 1a where almost equal rates of clinical cure and failure were observed in the subgroup of patients who had EOT antibody titers of < 1:320. Furthermore, no significant correlations were found between EOT antibody titers of either *Brucella* spp. and other outcomes except for EOT WBC count where lower *B. abortus* antibody titers were weakly associated with higher WBC counts (negative correlation) (R = − 0.43; *P* = 0.002). Interestingly, such correlation turned towards the opposite direction after the stratification based on the serological cure cutoff (R = 0.33; *P* = 0.002). Despite these findings, patients generally had normal mean WBC count at EOT. Subgroup analyses of patients with uncomplicated or complicated brucellosis showed similar results of lack of correlation between the EOT antibody titers and all the tested variables.

**Table 3:** Pearson’s correlation coefficient of the correlations between end of therapy *Brucella* antibody titers with different outcome variables (*n* = 63)

Variable	R with ***Brucella melitensis*** antibody titer (***P*** value)	R with ***Brucella abortus*** antibody titer (***P*** value)
Clinical cure	**− 0.29 (0.03)**^a^	**− 0.28 (0.03)**^a^
Mortality	−0.35 (0.79)	0.11 (0.39)
Length of stay	−0.26 (0.14)	− 0.27 (0.14)
Duration of therapy	−0.11 (0.42)	− 0.06 (0.68)
EOT temperature	0.01 (0.96)	0.01 (0.96)
EOT white blood cells count	−0.27 (0.06)	**−0.43 (0.002)**^b^
EOT C-reactive protein	−0.17 (0.29)	−0.14 (0.39)
EOT erythrocyte sedimentation rate	0.17 (0.34)	0.03 (0.88)

*EOT* End of therapy

^a^ This result indicates that lower antibody titers correlate with clinical cure; however, the results should be carefully interpreted given the small R^2^ values

^b^ This result indicates that the higher the antibody titers, the lower the white blood cells count. This result may not sound clinically reasonable given the correlation with clinical cure. However, the explanation behind this phenomenon is unknown

### Correlation with baseline parameters

Table 4 lists the correlation results of different baseline variables with baseline antibody titers. Despite the statistical significance of the correlation between the antibody titers of *B. abortus* with culture positivity, this correlation is considered weak. This because of the low value of the correlation coefficient (R = 0.29; *P* = 0.004), as well as the lack of difference in the rate of culture positivity among patients who met the serological diagnostic criterion at baseline as illustrated in Fig. 1b. Hence, the hypothesis that culture positivity correlates with high antibody titers is accepted. This finding persisted even after stratifying antibody titers based on the serological criterion for diagnosis of ≥1:640 (R = 0.3; *P* = 0.002).

**Table 4:** Pearson’s correlation coefficient of the correlations between baseline *Brucella* antibody titers with different baseline variables (*n* = 94)

Variable	R with ***Brucella melitensis*** antibody titer (***P*** value)	R with ***Brucella abortus*** antibody titer (***P*** value)
Age	−0.05 (0.65)	− 0.04 (0.71)
Location	0.04 (0.73)	0.2 (0.05)
Culture positivity	0.14 (0.17)	**0.29 (0.004)**^a^
Arthralgia	0.02 (0.86)	0.09 (0.4)
Complicated brucellosis	−0.09 (0.37)	**−0.25 (0.02)**^b^
Baseline temperature	0.07 (0.84)	−0.002 (0.99)
Baseline white blood cells count	0.03 (0.77)	−0.01 (0.92)
Baseline C-reactive protein	0.03 (0.79)	0.04 (0.74)
Baseline erythrocyte sedimentation rate	−0.001 (0.99)	−0.01 (0.97)

^a^ This result indicates that the higher the antibody titers, the higher the probability that a culture is positive in the 56.4% of the total patient population who had positive *Brucella* culture at baseline

^b^ This result indicates that the complicated brucellosis was associated with lower antibody titers at baseline. This result should be carefully interpreted given the low correlation coefficient

In addition to the lack of correlation between baseline antibody titers of either species with age and location, no correlations were found with the clinical manifestations of the disease. Although complicated brucellosis seems to correlate with a lower antibody titer at baseline as given by the significant *P* value, the correlation is considered null after it became insignificant when the antibody titers were divided into two groups based on the cutoff value of ≥1:640. In both cases, it should be noted that the correlation coefficient was low.

## Discussion

Brucellosis is an endemic disease in many regions worldwide [4, 5]. Given the endemic nature of the disease, only a few studies exist in the literature addressing the condition, and studies concerning antibodies and their relationship with disease outcomes are even scarce. Hence, this study aimed to fill the gap by assessing this relationship.

In a study of 116 clinically cured brucellosis patients, Almuneef, et al. found that *Brucella* antibodies remained detectable at EOT follow up despite full clinical recovery [8]. However, 2 years post completion of therapy, the antibody titers decreased dramatically and reached undetectable levels in some patients. A similar finding was seen in our study where about half of the patients who experienced clinical success did not achieve the serological success of antibody titers of < 1:320 at EOT. This fact is also supported by the lack of correlation between the antibody titers and clinical outcomes, including temperature and inflammatory markers (WBC count, CRP, and ESR). Nevertheless, it is presumed that *Brucella* antibody titers of those patients would eventually reach undetectable levels after 24 months or more post therapy. A similar finding was also reported by Roushan and colleagues where *Brucella* antibody titers remained detectable after 2 years in cured cases [12]. Moreover, Gazapo, et al. who used ELISA to measure *Brucella* IgG and IgM also reported measurable antibodies up to 13 months post diagnosis [13].

Our study also assessed the correlation of *Brucella* antibody titers with the clinical picture of brucellosis including the most common symptom of arthralgia, as well as fever, leukocytosis, and elevated inflammatory markers, CRP and ESR. A lack of correlation was demonstrated between *Brucella* antibody titers and these variables. These results resemble the findings from a study by Alsubaie and colleagues who evaluated the family members of brucellosis patients whether they appeared symptomatic or asymptomatic [14]. Symptoms sought in this study included arthralgia, fever, malaise, headache, anorexia, and weight loss. WBC counts and inflammatory markers were not assessed. Forty of the 178 family members screened for brucellosis had classic manifestations of the disease including arthralgia and fever, of whom only 18 (45%) had positive serology results (≥ 1:160). Thus, they were confirmed to have acute brucellosis (five had positive serology tests but claimed to have past infections). Such result further confirms the lack of correlation between brucellosis symptoms and serology test for *Brucella* as the remaining 55% symptomatic members had negative serology tests. This lack of correlation was also observed when only four (3%) of 138 members had positive serology tests despite being asymptomatic, hence they were diagnosed with the disease (seven had past infections with positive serology results).

While our study confirms previous findings of the lack of correlation of serologic results of *Brucella* with clinical outcomes, to our knowledge, this is the first study to evaluate the correlation between antibody titers and culture positivity. Since our institution is in an endemic country, patients are serologically diagnosed with brucellosis when antibody titers measure 1:640 or higher. However, when this criterion is not matched, a patient may still be diagnosed with the disease based on a positive blood culture for *Brucella* or classic clinical picture of the disease. As demonstrated in Fig. 1b, a positive serology is not always accompanied by a positive culture and vice versa. In the study by Alsubaie, et al., only eight (47.1%) of 17 seropositive patients had positive cultures for *B. melitensis* vs. none in the asymptomatic brucellosis group [14]. Both the findings of this study and the findings of our study indicate that serology testing for *Brucella* should not replace culturing, the gold standard testing for the disease. Furthermore, when serological results at baseline were assessed against the factors of patient’s age and hospital location in our study, a correlation was not observed.

## Conclusion

As studies on the relationship of serological test results of acute brucellosis with clinical outcomes and culture positivity are limited, this study provides evidence that *Brucella* serology does not correlate with clinical outcomes at EOT nor with culture positivity at baseline. Therefore, healthcare providers are advised to consider the complete clinical picture of a brucellosis patient, as well as culture, without relying solely on serological results.

## Data Availability

Data are available upon request from the authors.

## Abbreviations

CRP: C-reactive protein
ELISA: Enzyme-linked immunosorbent assay
EOT: End of therapy
ESR: Erythrocyte sedimentation rate
IQR: Interquartile range
MALDI-TOF: Matrix-Assisted Laser Desorption/Ionization-Time Of Flight
PCR: Polymerase chain reaction
SAT: Standard tube agglutination test
WBC: White blood cell

## References

[1] Tuon FF, Cerchiari N, Cequinel JC, Droppa EEH, Moreira SDR, Costa TP (2017). Guidelines for the management of human brucellosis in the state of Parana, Brazil. Rev Soc Bras Med Trop.

[2] Corbel MJ. Food and Agriculture Organization of the United Nations, World Health Organization & World Organisation for Animal Health. Brucellosis in humans and animals. Geneva: World Health Organization; 2006.

[3] Akhvlediani T, Bautista CT, Garuchava N, Sanodze L, Kokaia N, Malania L (2017). Epidemiological and clinical features of brucellosis in the country of Georgia. PLoS One.

[4] Dean AS, Crump L, Greter H, Schelling E, Zinsstag J (2012). Global burden of human brucellosis: a systematic review of disease frequency. PLoS Negl Trop Dis.

[5] Rubach MP, Halliday JE, Cleaveland S, Crump JA (2013). Brucellosis in low-income and middle-income countries. Curr Opin Infect Dis.

[6] Araj GF (2010). Update on laboratory diagnosis of human brucellosis. Int J Antimicrob Agents.

[7] Smith ME, Bhimji SS (2018). Brucellosis. StatPearls.

[8] Almuneef M, Memish ZA (2002). Persistence of *Brucella* antibodies after successful treatment of acute brucellosis in an area of endemicity. J Clin Microbiol.

[9] Al Dahouk S, Tomaso H, Nockler K, Neubauer H, Frangoulidis D (2003). Laboratory-based diagnosis of brucellosis--a review of the literature. Part I: techniques for direct detection and identification of Brucella spp. Clin Lab.

[10] Purwar S, Metgud SC, Mutnal MB, Nagamoti MB, Patil CS (2016). Utility of serological tests in the era of molecular testing for diagnosis of human brucellosis in endemic area with limited resources. J Clin Diagn Res.

[11] Memish ZA, Almuneef M, Mah MW, Qassem LA, Osoba AO (2002). Comparison of the *Brucella* standard agglutination test with the ELISA IgG and IgM in patients with *Brucella* bacteremia. Diagn Microbiol Infect Dis.

[12] Roushan MR, Amiri MJ, Laly A, Mostafazadeh A, Bijani A (2010). Follow-up standard agglutination and 2-mercaptoethanol tests in 175 clinically cured cases of human brucellosis. Int J Infect Dis.

[13] Gazapo E, Gonzalez Lahoz J, Subiza JL, Baquero M, Gil J, de la Concha EG (1989). Changes in IgM and IgG antibody concentrations in brucellosis over time: importance for diagnosis and follow-up. J Infect Dis.

[14] Alsubaie S, Almuneef M, Alshaalan M, Balkhy H, Albanyan E, Alola S (2005). Acute brucellosis in Saudi families: relationship between brucella serology and clinical symptoms. Int J Infect Dis.

